# Distinctive microbiota of delayed healing of oral mucositis after radiotherapy of nasopharyngeal carcinoma

**DOI:** 10.3389/fcimb.2022.1070322

**Published:** 2022-12-20

**Authors:** Rui Jiang, Yafang Liu, Han Zhang, Yitang Chen, Ting Liu, Jindi Zeng, Ermin Nie, Songling Chen, Jizhou Tan

**Affiliations:** ^1^ Department of Stomatology, The First Affiliated Hospital of Sun yat-sen University, Guangzhou, China; ^2^ Second Clinical Medical College, Guangzhou University of Chinese Medicine, Guangzhou, China; ^3^ Department of Stomatology, The First Affiliated Hospital of Guangzhou Medical University, Guangzhou, China

**Keywords:** oral mucositis (OM), radiotherapy, nasopharyngeal carcinoma, microbiota, 16S rRNA

## Abstract

**Background:**

Oral mucositis is the most common complication after radiotherapy of nasopharyngeal carcinoma (NPC). Previous studies had revealed that oral microbiota took great alteration soon after and during radiotherapy. Here, we aimed to investigate if the alteration of oral microbiota was related to delayed healing of oral mucositis after six month of radiotherapy.

**Methods:**

We recruited 64 NPC patients and collected samples after six month of radiotherapy. 32 patients were included into normal healing group (N), 22 patients were mild delayed healing group (M), while 10 patients were severe delayed healing group (S). 16S rRNA gene sequencing was used to assess and identify oral microbiota alteration.

**Results:**

The diversity of oral microbial communities was not significantly different. Composition of oral microbial was huge different among S group, for the *Actinobacteria* and *Veillonella* were significantly increased, which showed significant dysbiosis of the oral microbiome. Functional analysis of metabolic pathways of oral microbiota demonstrated that degradation of organic acids and amino acids were significantly increased in S group. Moreover, phenotype analysis found that relative abundance of aerobic and biofilm formation were higher in S group. We also found the *Actinobacteria* co-occurred with *Veillonellaceae*, but anti-occurred with other biofilm oral bacteria. These two biomarkers may be predictable for severe delayed healing of oral mucositis after radiotherapy.

**Conclusion:**

This study suggests a potential association between oral microbiome and delayed healing of oral mucositis. The *Actinobacteria* and *Veillonellaceae* may be biomarkers in predicting the risks for the severe delayed healing of oral mucositis after radiotherapy of NPC.

## Introduction

Nasopharyngeal Carcinoma (NPC) is one of the most common cancers occurring in Southeast Asia and South China. In 2020, the number of new cases of NPC reached nearly 133,354, with China accounting for 62,444 cases (46.8%). The estimated number of nasopharyngeal cancer-related deaths was 80,000, with China accounting for nearly 34,810 (43.5%) (Siak et al., 2021). Radiotherapy is the preferred treatment strategy for patients with nasopharyngeal carcinoma. The common oral complications after radiotherapy include radiation-induced oral mucositis, parotid gland injury, jaw necrosis, osteomyelitis, and dental caries (Sroussi et al., 2017; Baudelet et al., 2019; Rao et al., 2021). Radiation induced oral mucositis (RIOM) is the damage of oral mucosa caused by radiation. RIOM is divided into acute injury and chronic injury according to the duration of radiotherapy.

Chronic oral mucositis was the main complication more than 3-6 months after radiotherapy of NPC, which mainly caused great pain to NPC patients (Buechler et al., 2021; Tuominen and Rautava, 2021). Oral mucositis is typically characterized by erythema, edema, mucosal ulceration and pseudomembrane formation in the mouth and oropharynx. Patients with oral mucositis lesions often complain of severe pain and dysphagia. Severe mucositis not only affects the patient’s quality of life, but also increases the response to total parenteral nutrition, interruption of cancer treatment, and enhances risk of infections (Trotti et al., 2003; Stokman et al., 2006; Villa and Sonis, 2015). Although many patients can have a total healing of oral mucositis after 6 month later of radiotherapy, there are still some patient who had a very slow healing of mucosal injury and had long-term of pain after 6 month of radiotherapy. The underling mechanisms of which needs to be furtherly studied.

Many studies has revealed that oral microbiota had great change during or soon after radiotherapy for NPC, which were related with oral mucositis. It may promote oral mucositis development through inflammatory pathways and the interference in the energy balance of mucosal cells (Zhu et al., 2017; Hou et al., 2018; Irfan et al., 2020). However, all the results were concentrated on the alteration of oral mucositis during the procedure of radiotherapy or soon after radiotherapy. They only focused on the oral mucositis at early stage, but neglected the delayed healing of oral mucositis after six months of radiotherapy. It will have more clinical significance, if the changes of oral microbiota can be used to predict the delayed healing of oral mucositis after six months of radiotherapy. Meanwhile, Manly researches had verified that oral mucositis could be a biomarker to predict the cancer progression and severity of complications. For example, one study showed that oral microbiome has potential implications for the early detection and prevention of esophageal cancers (Peters et al., 2017). Another study revealed that oral microbiome dysbiosis was a novel non-invasive biomarker in detection of colorectal cancer (Zhang et al., 2020). However, few studies have focused on the potential of prediction of the oral microbiota profiles between patients with normal healing or severe delayed healing of oral mucositis after six months of radiotherapy.

In this study, we hypothesize that the severe delayed healing of oral mucositis of NPC radiotherapy may have an underlying microbial basis. We enrolled 64 NPC patients who took radiotherapy, and divided them into severe delayed healing (S) group, mild delayed healing (M) group, and normal healing (N) group into N group. We further explored this potential findings in the quest for novel non-invasive biomarkers for predicting severe delayed healing of oral mucositis after NPC radiotherapy.

## Methods

### Participant recruitment

The recruited patients were newly diagnosed with nasopharyngeal carcinoma by the Department of Otolaryngology, The First Affiliated Hospital of Sun Yat-sen University from April 2021 to September 2021. The patients and their families were aware of the study methods and obtained written consent. The present study protocol was approved by the ethics board of The First Affiliated Hospital of Sun Yat-sen University. Finally, 64 NPC patients were recruit in this study.The inclusion criteria were as follows:

1) Aged from 20 to 80 years old

2) Cancer clinical stage I - IV

3) WHO pathological type II-III

The exclusion criteria were as follows:

1) A history of antibiotic use in the past 3 month.

2) A history of radiotherapy or chemotherapy.

3) Severe periodontitis or dental caries.

4) Already have oral mucositis or recurrent attacks

All patients received VMAT radical radiotherapy at follows. GTV (gross target volume): 68.1Gy/30Fr; CTV1 (Clinical target volume 1/high-risk area): 60Gy/30Fr; CTV2 (Clinical target volume 2/low risk area): 54Gy/30Fr. All patients also received whole course concurrent chemotherapy (Docetaxel 60mg/m^2^+Cis-platinum 60mg/m^2^). Six month later after the chemotherapy and radiotherapy were down, all patients go to department of stomatology for sample collection. Samples were obtained directly from the mucosal surface of the soft palate by throat swab, especially at the surface of injury mucosa. Before sampling, patient rinsed mouse with water without disinfection mouthwash. The contact time with air was as short as possible, and the samples were flushed into the EP tube with sterile normal saline. The tubes were immediately transferred to the ultra-low temperature refrigerator at -80°C in the laboratory.

The evaluation of scoring scales of the severity of oral mucositis is based on WHO scale and OMAS scale, followed by reference (Hong et al., 2019). WHO scale evaluates in a categorical scale of 0 to 4 objective signs and patientreported symptoms. Patients of 0 score represent Normal healing (N) group. Patients of 1 to 2 score represent M group, and patients of 3 to 4 score represent S group. The OMAS scale is based solely on objective signs of erythema and ulceration. OMAS scores reported here could range from 0 to 45 and represent the aggregated scores from nine intra-oral sites evaluated. For the power calculation, the balanced one-way analysis of variance power calculation was achieved using SPSS and R package “pwr”. The statistical power value = 81.92% for SPSS, and 88.28% for R package. Hence, the sample numbers in each group were sufficient.

### DNA extraction and 16S rRNA gene amplicon sequencing

Microbial DNA was extracted from oral samples using the HiPure Stool DNA Kits (Magen, Guangzhou, China), and the following protocol was based on the manufacturer’s instructions. PCR amplification of the V3-V4 region of the 16S rRNA gene was performed under conditions of denaturation. Primers 341F: CCTACGGGNGGCWGCAG and 806R: GGACTACHVGGGTATCTAAT were used to amplify the 16S rRNA gene fragment. For amplicon sequencing, libraries were prepared with MiSeq library preparation Reagent Kit v3 (New England Biolabs, USA) and then sequenced on a NovaSeq 6000 Sequencer platform (Illumina, USA).

### Data preparation and analysis

For data filtering, FASTP (version 0.18.0) was used to filter raw data from the Illumina platform. For sequence splicing, FLASH software (version 1.2.11) was used to combine clean reads into tags according to the threshold of minimum overlap of 10bp and maximum mismatch rate of 2%. Noisy sequences of raw tags were filtered under specific filtering conditions (Edgar, 2013) to obtain the highquality clean tags. Filled tags were clustered into Operational taxonomic units (OTUs) according to ≥97% similarity using UPARSE(version 9.2.64) software (Bolyen et al., 2019). All chimeric tags were removed using UCHIME algorithm (Quast et al., 2013) and finally obtained effective tags for further analysis. The tag sequence with highest abundance was selected as representative sequence within each cluster.

Alpha diversity analysis was calculated in QIIME software(version 1.9.1). The exponential dilution curve of diversity and rank-abundance curve were drawn using GGPlot 2 package in R language. Beta diversity analysis was performed using Muscle (version 3.8.31) for OTU representative sequence alignment. Multivariate statistical techniques including PCoA (principal coordinates analysis) of (Un) weighted unifrac, jaccard and bray-curtis distances were generated in R project Vegan package (Edgar et al., 2011) (version 2.5.3) and plotted in R project ggplot2 package (Nilsson et al., 2018) (version 2.2.1). To indicate species analysis, Venn Diagram package in R language was used for Venn analysis, and the R language UpSet R package was used to analyze shared unique species/OTUs between groups. Linear discriminant analysis (LDA) effect size (LEfSe) analysis was performed to identify taxa showing the most significant differences in microbial abundance between groups (Segata et al., 2011). Only taxa with LDA scores > 2.0 and *p* < 0.05 are shown. KEGG (Kyoto Encyclopedia of Genes and Genomes) metabolic pathway analysis of sample bacteria or archaea was performed using PICRUSt (Parks et al., 2014).

### Statistical analysis

Associations between the clinical characteristics were performed by Pearson’s Chi-square test or Fisher’s exact test. The *p* value less than 0.05 were considered as statistically significant (*, <0.05; **, <0.01; ***, <0.001). The Turkey-HSD analysis was performed to analyze the alpha-diversity differences. In LEfSe analysis, phenotypic prediction Bugbase was revealed by contribution stacking diagram and analyzed by Turkey-HSD test, *P <*0.05, with * for significant correlation. Microbiome phenotype analysis was used by BugBase, and tested by Pearson’s Chi-square test (*, <0.05; **, <0.01; ***, <0.001). All data were analyzed using GraphPad Prism v. 6.01, and R package v. 3.3.2 (Xia et al., 2018).

## Results

### Clinical characteristics of patients after radiotherapy of nasopharyngeal carcinoma

**Figure 1:**
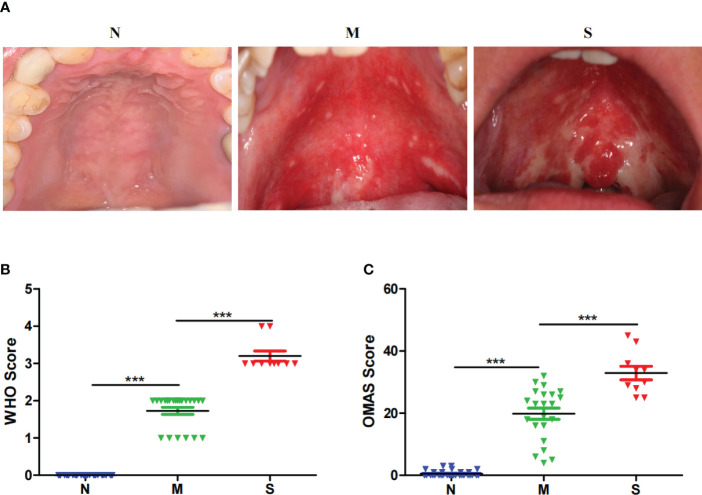
Clinical presentation of oral mucosal injury after the radiotherapy of nasopharyngeal carcinoma. **(A)** Intra-oral images of oral mucosal injury of different patients in normal healing group (N), severe delayed healing (S) and mild delayed healing (M) groups. The grouping scheme is based on the WHO scoring criteria, which evaluates in a categorical scale of 0 to 4 objective signs and patient reported symptoms. Patients of 0 score represent Normal healing (N) group. Patients of 1 to 2 score represent M group, and patients of 3 to 4 score represent S group. **(B)** The clinical scores of mucosal injury in S group (n=10), M group (n=22) and N group (n=32) for the WHO criteria. **(C)** The clinical scores of mucosal injury in S group (n=10), M group (n=22) and Normal group (n=32) for the OMAS criteria. The OMAS scale is based solely on objective signs of erythema and ulceration. OMAS scores reported here could range from 0 to 45 and represent the aggregated scores from nine intra-oral sites evaluated. ***indicates a *p* value < 0.001 when comparing each groups *via* Wilcoxon matched-pairs signed rank tests.

A total of 64 cases were recruited in this study. All samples were collected at the six months after radiotherapy and chemotherapy. The patients were divided into three sub-groups based on the healing degree of oral mucositis. Patients with severe delayed healing were divided into S group, and patients with mild delayed healing were divided into M group, and patients with normal healing were divided into N group. Typical injury presentations of erythema and ulceration were mostly on the non-keratinized mucosa, such as soft palate and pharyngeal area (**Figure 1A**). The severity of oral mucosal injury was based on WHO score scales (Hong et al., 2019) (**Figure 1B**). Another commonly used scoring scale called OMAS, was also used to evaluate the severity of oral mucosal injury among two groups (**Figure 1C**). There was no significant differences in the clinical characteristics, including age, gender, BMI, alcohol drinking, smocking, diabetes status among the three groups (**Table 1**).

**Table 1:** Clinical characteristics of the enrolled participants.

Characteristics	Normal healing (N, n = 32)	Severe delayed healing (S, n = 10)	Mild delayed healing (M, n = 22)	*p* value
Age (mean ± SD)	49.3 ± 11.25	46.6 ± 9.58	52.5 ± 8.72	0.279^*^
BMI (mean ± SD) kg/ m2	22.74 ± 1.78	22.15 ± 2.04	22.56 ± 2.15	0.446^*^
Sex male	17 (53.1%)	5 (50.0%)	15 (68.2%)	0.324^#^
Smoking	19 (59.4%)	4 (40.0%)	9 (40.9%)	0.283^#^
Alcohol drinking	10 (31.3%)	3 (30.0%)	8 (36.4%)	0.906^#^
Diabetes	9 (28.1%)	2 (20.0%)	6 (27.3%)	0.875^#^
TNM stage				0.147^#^
Stage I	0 (0.0%)	0 (0.0%)	0 (0.0%)	
Stage II	15 (46.8%)	3 (30.0%)	11 (50.0%)	
Stage III	11 (34.3%)	2 (20.0%)	6 (27.3%)	
Stage IV	6 (18.7%)	5 (50.0%)	5 (22.7%)	
Prognosis				0.882^#^
CR	1 (3.1%)	0 (0.0%)	0 (0.0%)	
PR	6 (18.7%)	2 (20.0%)	4 (18.2%)	
SD	19 (59.3%)	7 (70.0%)	12 (54.5%)	
PD	6 (18.7%)	1 (10.0%)	6 (27.2%)	

^*^Tested by One-way analysis of variance (ANOVA).

^#^Tested by Pearson’s chi-square (x^2^) test.

### Bacterial diversity of the oral microbiota of delayed healing of mucositis

**Figure 2:**
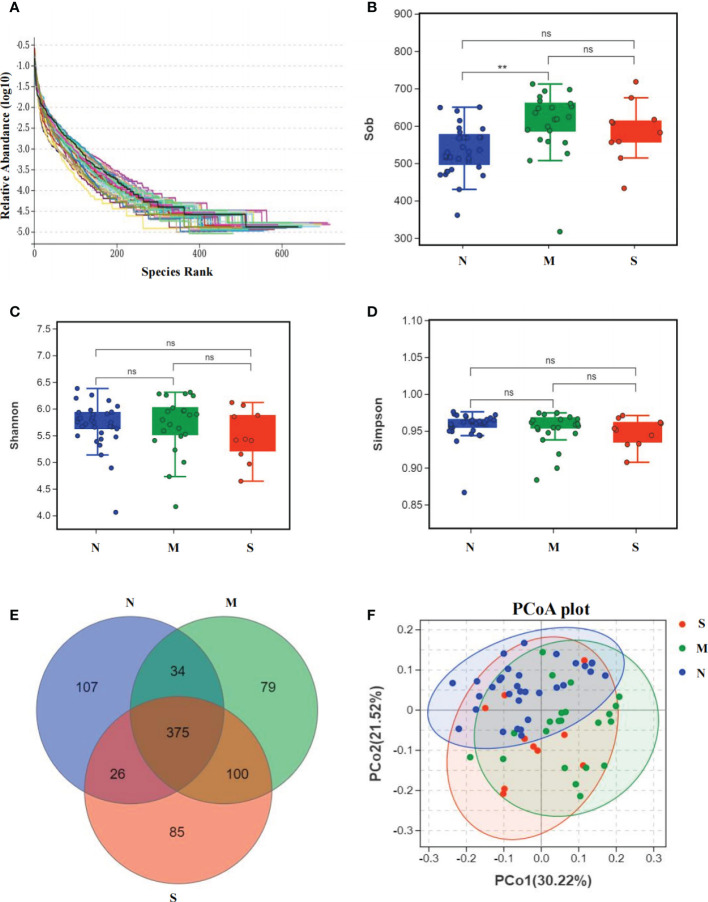
Bacterial diversity of the oral microbiota collected from nasopharyngeal carcinoma patients. **(A)** The relative bacterial evenness of each sample was evaluated by the rank abundance curves. **(B-D)** α-diversity of oral microbial were estimated by the Sob index, Shannon index and Simpson index. Turkey-HSD analysis was used. ** = *p* value < 0.01, ns = no significant difference. **(E)** The Venn diagram displayed the overlaps between all three groups based on OTUs level. **(F)** β-diversity was calculated using weighted UniFrac by PCoA plot. Kru-Wall test was used, and *p* value=0.64.

The oral microbiota was assessed by 16S rRNA gene MiSeq sequencing. The relative bacterial evenness was evaluated by rank abundance curves, which exhibited similar patterns in all samples (**Figure 2A**). Alpha-diversity was used to calculate and evaluate the differences in bacterial diversity among the three groups, which was analyzed on OTU level and revealed by Sob, Shannon and Simpson index. The results showed that the Alpha-diversity of S and M groups were higher than N group based on Sob index, but was no significant difference between S and M groups. Based on Shannon and Simpson index, three was no significant difference among three groups (**Figures 2B-D**). In addition, the Venn diagram revealed the 375 of the total 806 OTUs were shared among the three groups. Notably, 85 OTUs were unique for the S group (**Figure 2E**). To reveal the microbiome space between three samples, beta-diversity was calculated by the unweighted UniFrac method of the principal coordinate nanlysis (PCoA) based on OTU level. The results showed that the alpha-diversity and beta-diversity of oral microbial communities in these three groups were not significantly different (**Figure 2F**).

### Comparison of oral microbial communities in S and M groups

**Figure 3:**
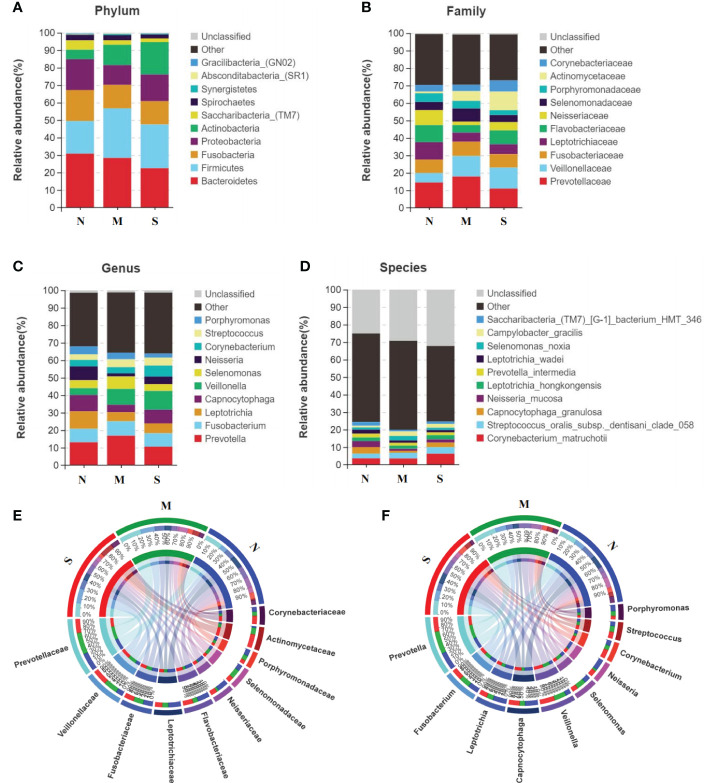
Oral microbiota composition of all three groups. **(A-D)** Average composition of bacterial community at the phylum, family, genus and species levels represent the top 10 dominant species. **(E, F)** Circos circle diagram was used to show the corresponding relationship between sample groups and species, reflecting the composition proportion and distribution of dominant species among different groups at family level **(E)** and genus level **(F)**.

The average composition of oral bacterial was shown at phylum, family, genus, and species levels respectively. At the phylum level, *Bacteroidetes, Firmicutes, Fusobacteria, Proteobacteria*, and *Actinobacteria* were the top five dominant bacterial phyla in the three groups. *Actinobacteria* was increased in the S group than M group, and was also added in S group than N group (**Figure 3A**). At the family level, *Prevotellaceae, Veillonellaceae, Fusobacteriaceae, Leptotrichiaceae, and Flavobacteriaceae* were the top five bacterial family in three groups, and *Veillonellaceae* was significantly enriched in S group than M and N groups. In addition, *Neisseriaceae* was decreased in S and M groups than N group. Most importantly, *Actinomycetaceae* was significantly added in S group than M and N groups (**Figure 3B**). At the genus level, *Prevotella, Fusobacterium, Leptotrichia, Capnocytophaga*, and *Veillonella* were the top five genus in three groups, and *Veillonella* was significantly increased in S group than N group (**Figure 3C**). At the species level, *Leptotrichia_hongkongensis, Neisseria_mucosa, Capnocytophaga_granulosa, Streptococcus_oralis_subsp_dentisani_clade_058, Corynebacterium_matruchotii* were the top five species in three groups (**Figure 3D**). The Circos circle diagram also showed the tendency, reflecting the composition proportion and distribution of dominant microbial communities of S group was different from another two groups at family and genus level (**Figures 3E, F**).

### Functional analysis of oral microbiota in S and M groups

**Figure 4:**
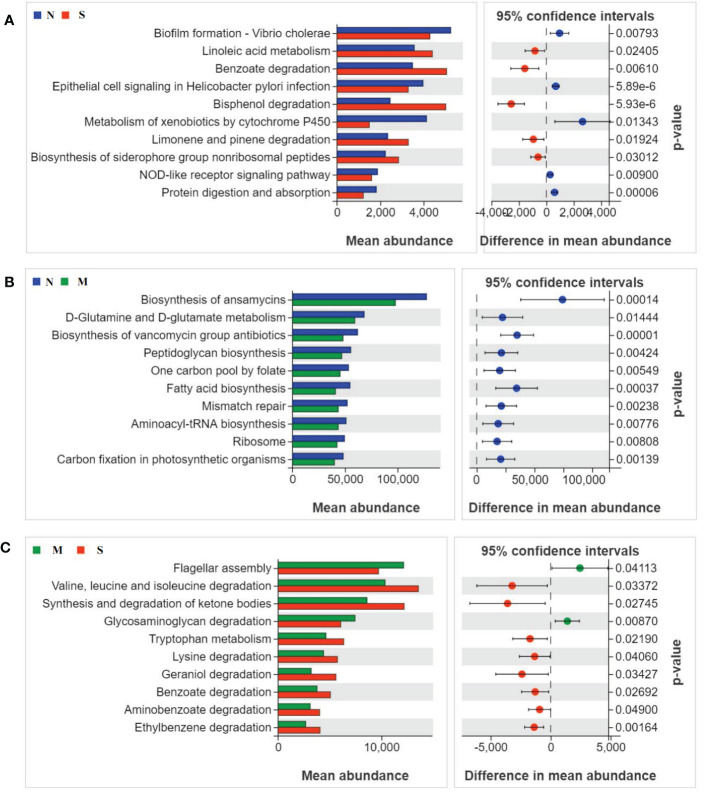
Oral microbial functional dysbiosis in metabolism pathways. Differential KEGG pathways were analyzed using PICRUSt 2, and PCoA analysis was conducted for the three groups. The top 10 significant differences between S group and N group **(A)**, M group and N group **(B)**, and S group and M group **(C)** were presented respectively.

To study the function and metabolism changes in oral microbial communities, the Phylogenetic Investigation of Communities by Reconstruction of Unobserved States (PICRUSt) was used to analyze all OTUs. The PICRUSt analysis identified top ten KEGG pathways in significant differences among all three groups (**Figures 4A-C**). The pathways of metabolism of degradation of organic acids and amino acids were significantly increased in S group compared with those of M group and N group, such as benzoate and valine degradation. In contrast, the pathways involved in glycosaminoglycan degradation, biofilm formation, flagellar assembly, and metabolism of xenobiotics by cytochrome P450 were inhibited in S groups related to thosed in M and N groups. In addition, pathways of D-glutamine metabolism, fatty acid biosynthesis, and aminoacyl-tRNA biosynthesis were down-regulated in M group compared with N group.

### Phenotype analysis of oral microbiota all three groups

**Figure 5:**
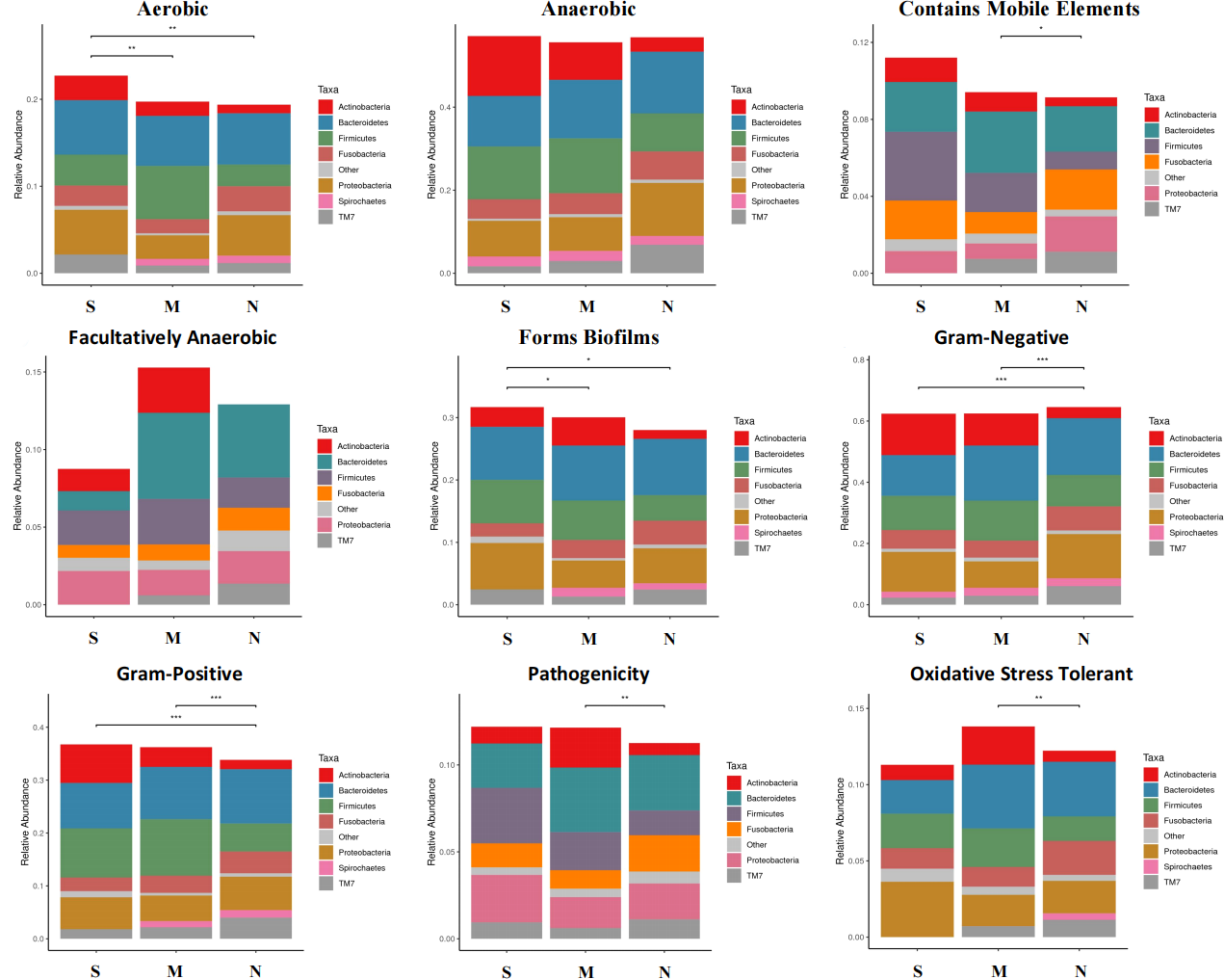
Microbial phenotype analysis of all three groups. The BugBase analysis was used to reveal the characteristics of the microbial phenotype of all groups, which was divided into nine phenotypic classifications. The above figure shows the relative abundance and comparison of bacterias with different groups in different phenotypes. Turkey-HSD analysis was used. *p value< 0.05, **p value< 0.01, ***p value< 0.001.

To study the difference of phenotype of oral microbiota among three groups, we used the BugBase analysis to reveal the characteristics of the microbial phenotype of all groups, which were divided into nine phenotypic classifications. We found that in the aerobic phenotype, the relative abundance of bacteria in S groups was significantly higher than those in M group and N group. Similarly, the relative abundance of bacteria of S group was also higher than those in M and N groups in the phenotype of biofilm formation. In addition, we also found that the relative abundance of both S and M groups were lower than N group in gram-negative phenotype, while that of S and M groups were higher than N group in gram-positive phenotype. We did not found significant difference between S and N groups in the phenotypes of anaerobic, contains mobile elements, facultatively anaerobic, pathogenicity, and oxidative stress tolerant. These results showed the severe delayed healing group had higher bacteria abundance level of aerobic, biofilm formation, and gram-positive, but lower abundance level of gram-negative phenotype (**Figure 5**).

### Comparison of the composition and correlation network analysis of oral microbiome in S and M patients

**Figure 6:**
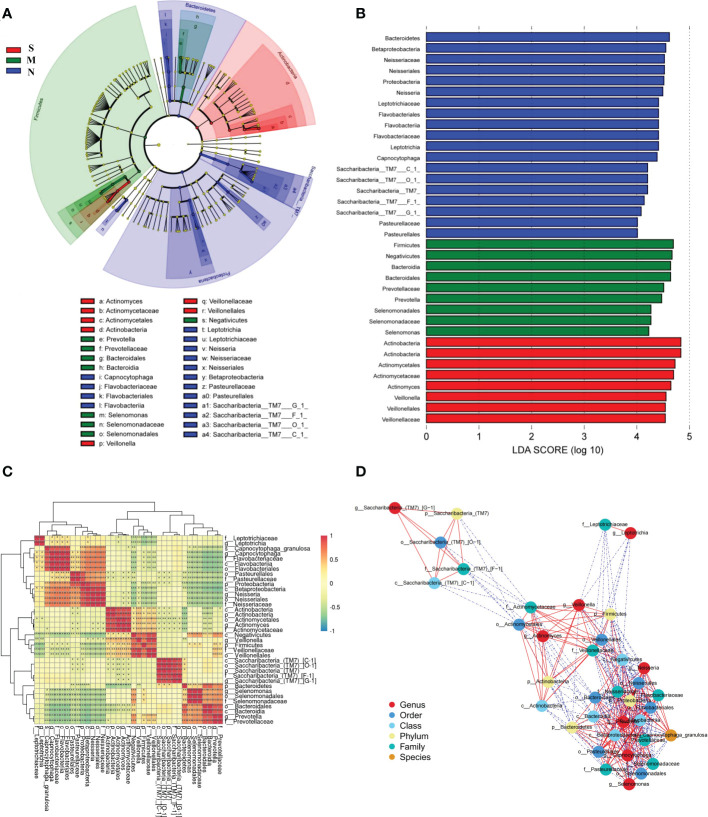
The specific oral microbial differences and their correlation among all three groups. **(A)** The analysis of LEfSe reveals the predominant microbes in different groups. The color represents the respective group. **(B)** The bar chart above shows the microbes with significant differences whose LDA score is greater than the *p* value (Only the taxa meeting a significant LDA threshold value of >4 are shown), indicating that the biomarker had statistical differences. **(C)** Heatmap showing Spearman correlation coefficients of the selected biomarkers in **(A, B)**. **(D)** Bacterial co-occurrence and anti-occurrence were investigated and presented as a network. The nodes representing core bacterial genera (colored according to different levels), and edges representing interactions (red=co-occurrence, blue=mutual exclusion) at *p* < 0.05. Turkey-HSD analysis was used. *p value< 0.05, **p value< 0.01, ***p value< 0.001.

LEfSe analysis was used to determine and distinguish the composition of oral microbiome among all three groups. The phylogenetic tree showed the phylogenetic relationships among the three groups, and a cladogram map was drawn to show the distribution of the dominant groups (**Figure 6A**). The different taxa were further extracted and shown on the bar chart, and only when the LDA score was greater than the preset value of 4, there were significant species differences among the three groups (**Figure 6B**). The length of the bars indicated the degree of impact of species that differed significantly among three groups. The results revealed 8 taxa of the oral microbiome, including *Actinobacteria* and *Veillonellaceae*, which were extremely enriched in the S group. For the M group, 9 kinds of microbial biomarkers, including *Firmicutes, Negativicutes, Bacteroidia, Prevotella*, and *Selenomonas*, were the predominant flora. For the N group, there were 19 taxa of bacterial markers were accumulated, including *Neisseria, Flavobacteriia*, and *Saccharibacteria_TM7.*

There was significant dysbiosis of the oral bacterial microbiome in S groups, as shown by differences in bacterial composition, diversity, and function among the three groups. So we focused on the selected 36 bacterial biomarkers above and clustered them based on the abundance profiles (**Figure 6C**). We identified the one pathogen oral bacterial co-abundance groups (pCAGs) (e.g. *Actinobacteria* and *Veillonellaceae*), and two kinds of biofilm oral bacterial co-abundance groups (bCAGs) (e.g. *Neisseria, Bacteroidia, Prevotella*, and *Flavobacteriia*), (e.g. *Bacteroidia, Prevotella*, and *Selenomonas*). It indicated that the oral bacterial microbiome in S groups had greater difference and less connection between M and N groups. The connection of oral bacterial microbiomes between M and N groups were more closed.

In addition, a stringent network analysis was performed to obtain further insights with respect to correlative bacterial populations at the all levels. It reveals that the apparent dissimilarity of the microbial composition when comparing the three groups. The resulting network showed significant co-occurrence and anti-occurrence (**Figure 6D**). The oral pathogen of 5 kinds of *Actinobacteria* co-occurred with 3 kinds of *Veillonellaceae*, and anti-occurred with other biofilm oral bacteria. In the bCAGs groups, nearly all the bacteria were co-occurred with others. We also found the *Saccharibacteria_TM7 were independent and had limited connection with other bacteria.*

**Figure 7:**
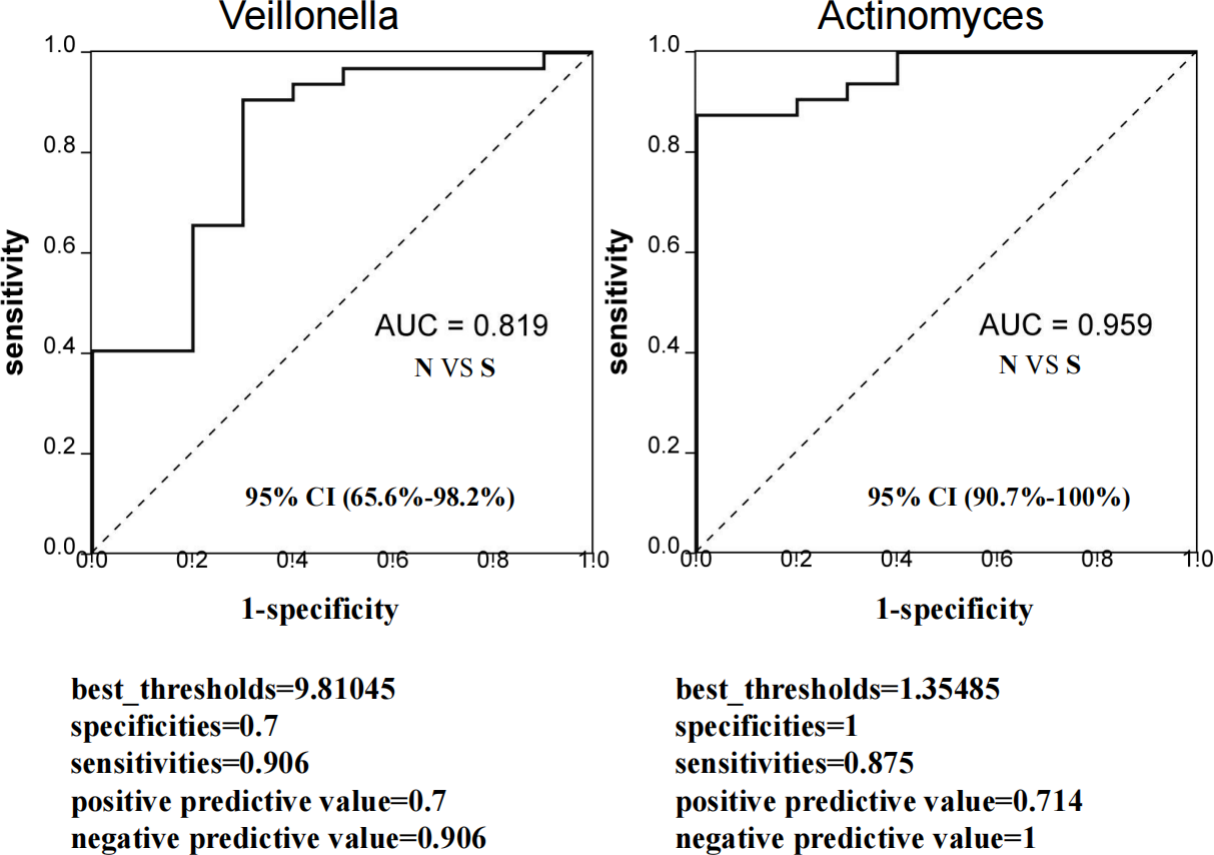
Evaluation of predictive ability of sellected microbiota biomarkers for severe delayed healing group. ROC curve represent the predictive ability of *Veillonella* and *Actinomyces* for S group. If AUC score is between 0.7 and 0.9, it represents a high accuracy. If AUC=0.5, it indicates that the diagnostic method is completely ineffective and has no diagnostic value. All samples including 10 S group and 32 N group were used to evaluate AUC values. The AUC value of *Veillonella* was 0.819 (95% CI: 65.6%-98.2%), and AUC value of *Actinomyces* was 0.959 (95% CI: 90.7%-100%). The best_thresholds, specificities, sensitivities, positive/negative predictive values were also revealed.

To clarify if the Veillonella and Actinomyces can be predictable markers of severe delayed healing of oral mucositis, we established the ROC curves to study the predictive ability of Veillonella and Actinomyces. The results indicates these two diagnostic biomarkers is effective and has diagnostic value (**Figures 7A, B**).

## Discussion

Oral mucositis is the most common toxicities of NPC radiotherapy. The severity of radiation-induced mucositis is correlated with the cumulative dose and duration of radiotherapy, especially at the end of radiotherapy, when the cumulative dose reached the highest, the degree of oral mucosa inflammation caused by radiotherapy was the most serious (Rodríguez-Caballero et al., 2012; Xu et al., 2014; Vanhoecke et al., 2015). Virtually, nearly all the patients experienced some degree of mucositis, and chemoradiotherapy has been shown to impair oral defense mechanisms and cause significant changes in the oral microbiota. In this study, the cumulative dose of all the patients was mainly divided into three types: primary tumor area (GTV)68.1Gy/30Fr, high-risk subclinical lesion area (CTV1)60Gy/30Fr and low-risk subclinical lesion area (CTV2)54Gy/30Fr. There was no significant difference in cumulative dose between each patient. A total of 30 radiotherapy sessions were performed for each patient, and the average radiotherapy duration was 6 weeks. There were no significant differences in the cumulative dose and duration of radiotherapy among all patients. The oral mucosa WHO and OMAS score grading assessment was performed at six month later after radiotherapy for classifying patients into groups (Hong et al., 2019).

In alpha-diversity analysis, we used Observed_species index (Sob index), Shannon index, and Simpson index to calculate the number of detected OTUs to represent the species abundance. On the whole, the abundance of oral microbial community did not changed a lot between different groups, which meant the delayed healing of oral mucositis did not affect the alpha-diversity of oral microbial community. The results was different with previous studies, which collected patient samples during the radiotherapy or within 30 days of radiotherapy (Zhu et al., 2017; Hou et al., 2018). The possible reason is that the oral mucosa microbiota gradually recovers 5-6 months after the end of radiotherapy and is very close to the situation before treatment, while radiotherapy will have a great impact on the oral microbiota in the short term. Hence, the differences of alpha-diversity analysis between different groups were not significant.

The species composition of oral microbiota at all levels were observed among three groups. The correlation between the composition of oral microbiota and the degree of oral mucosal injury after radiotherapy was evaluated by observing the trend of bacterial species composition. Firstly, we found *Actinobacteria* in the phylum and family levels were increased in severe delayed healing group, and *Veillonella* was also added. It reported that the higher abundance of *Actinobacteria* and *Fusobacteria*, the more likely the severity of oral mucositis is (Yamashita and Takeshita, 2017). Oral bacterial diversity has been reported to be higher in oral diseases, such as periodontal disease (Lamont et al., 2018). The *Actinobacteria* are commonly found as oropharyngeal symbioses and opportunistic pathogens that are commonly involved in the pathogenesis of meningitis, sinusitis, pleural empyema, and bronchopneumonia in patients with associated underlying diseases (Feder, 1990). It may profoundly affect oropharyngeal microbial homeostasis and is one of the related factors that predispose patients to severe mucositis, especially when the host immune system is weak (Sato et al., 2012; Shimada et al., 2012). *Veillonella* is associated with dental caries, periodontal disease, pulp and periapical disease, halitosis and other oral diseases*. Veilloncoccus* may contribute to the adhesion of *Streptococcus_mutans* and can decompose the lactic acid produced by *streptococcus_mutans* metabolism and provide adhesion sites for *porphyromonas gingivalis* and participate in the occurrence and development of periodontal disease by promoting immune response (Periasamy and Kolenbrander, 2010; Washio et al., 2014; Do et al., 2015).

In this study, we further analyzed the species function of oral bacteria by PICRUSt analysis, and observed the correlation between the species function and the degree of oral mucositis after radiotherapy. The metabolism and degradation of organic acids and amino acids were significantly increased in severe delayed healing group. In fact, studies showed the *Actinobacteria* and *Veillonella* in severe delayed healing group were enriched*. Veillonella* cannot metabolize carbohydrates and polyols, but can convert short chain organic acids, especially lactic acid, into less acidic acetic acid and propionic acid (Konings et al., 1975; Do et al., 2015). *Veillonella* can also produce H_2_S by degrade L-cysteine (Washio et al., 2005). Meanwhile, *Actinobacteria* can also affect the metabolisms of amino acids and acidic acetic acid, which may cause the delayed healing of mucosal injury (Sato et al., 2012; Shimada et al., 2012). Interestingly, we found that *Actinobacteria* and *Veillonella* were both opportunistic pathogens and showed significant co-abundance relationship. What’s more, both *Veillonella and Actinomyces* can be predictable markers of severe delayed healing of oral mucositis. However, we failed to develop a random forest model to predict this hypothesis, which may be mainly due to the minor differences between the subgroups and the insufficient sample size. Therefore, larger prospective cohort studies are needed to validate and validate this prediction model.

Clinically, some commonly used broad-spectrum antiseptic drugs or antimicrobials often fail to prevent the occurrence of severe mucositis (Wijers et al., 2001; Stokman et al., 2003; Nicolatou-Galitis et al., 2013). This is because untargeted antibiotic therapy may aggravate the dysregulation of microbial homeostasis in the early stage of oral mucositis after radiotherapy, and then the dysregulation of microbial homeostasis in the later stage of healing may cause the accelerated reproduction of opportunistic pathogens and aggravate inflammation. Therefore, it can be inferred that the key to prevent severe oral mucositis and accelerate its healing is to use specific antibiotics to suppress *Veillonella and Actinomyces*, and suppress inflammation in the late healing stage of oral mucositis.

In conclusion, our findings suggest that changes in oral microbiota are associated with the delayed healing of radiotherapy-induced mucositis in NPC patients, and that microbiota based strategies can be used to early predict and cure for severe mucositis after radiotherapy.

## Data availability statement

The data presented in the study are deposited in NCBI repository, accession number PRJNA907138. 

## Author contributions

RJ and JT designed experiments, RJ, YL, YC performed experiments, HZ, TL, JZ, EN, SC analyzed and interpreted the data. YC, TL, JZ, EN collected clinical samples and data. RJ and JT and wrote the manuscript with the revision. JT supervised the project. All authors contributed to the article and approved the submitted version.

## References

[B1] Baudelet M. Van den Steen L. Tomassen P. Bonte K. Deron P. Huvenne W.. (2019). Very late xerostomia, dysphagia, and neck fibrosis after head and neck radiotherapy. Head Neck 41 (10), 3594–3603. doi: 10.1002/hed.25880 31329343

[B2] Bolyen E. Rideout J. R. Dillon M. R. Bokulich N. A. Abnet C. C. Al-Ghalith G. A.. (2019). Reproducible, interactive, scalable and extensible microbiome data science using QIIME 2. Nat. Biotechnol. 37 (8), 852–857. doi: 10.1038/s41587-019-0209-9 31341288 PMC7015180

[B3] Buechler M. B. Fu W. Turley S. J. (2021). Fibroblast-macrophage reciprocal interactions in health, fibrosis, and cancer. Immunity 54 (5), 903–915. doi: 10.1016/j.immuni.2021.04.021 33979587

[B4] Do T. C Sheehy E. Mulli T. Hughes F. Beighton D. (2015) Transcriptomic analysis of three veillonella spp. present in carious dentine and in the saliva of caries-free individuals. Front. Cell Infect. Microbiol. 5 (25). doi: 10.3389/fcimb.2015.00025 PMC4374535 25859434

[B5] Edgar R. C. (2013). UPARSE: Highly accurate OTU sequences from microbial amplicon reads.Nat. Methods 10 (10), 996–998. doi: 10.1038/nmeth.2604 23955772

[B6] Edgar R. C. J Haas B. Clemente J. C. Quince C. Knight R. (2011). UCHIME improves sensitivity and speed of chimera detection. Bioinformatics 27 (16), 2194–2200. doi: 10.1093/bioinformatics/btr381 21700674 PMC3150044

[B7] Feder H. M. (1990). Actinomycosis manifesting as an acute painless lump of the jaw. Pediatrics 85 (5), 858–864. C.OMMAJ.R.X.X.X. doi: 10.1016/0022-3468(91)90454-2 2109853

[B8] Hong B. Y. Sobue T. Choquette L. Dupuy A. K. Thompson A. Burleson J. A.. (2019). Chemotherapy-induced oral mucositis is associated with detrimental bacterial dysbiosis. Microbiome 7 (1), 66. doi: 10.1186/s40168-019-0679-5 31018870 PMC6482518

[B9] Hou J. Zheng H. Li P. Liu H. Zhou H. Yang X. (2018). Distinct shifts in the oral microbiota are associated with the progression and aggravation of mucositis during radiotherapy. Radiotherapy and Oncology 129, 1, 44–51. doi: 10.1016/j.radonc.2018.04.023 29735410

[B10] Irfan M. Delgado R. Frias-Lopez J. (2020). The oral microbiome and cancer. Front. Immunol. 11. doi: 10.3389/fimmu.2020.591088 PMC7645040 33193429

[B11] Konings W. N. Boonstra J. De Vries W. (1975). Amino acid transport in membrane vesicles of obligately anaerobic veillonella alcalescens. J. Bacteriol 122 (1), 245–249. doi: 10.1128/jb.122.1.245-249.1975 164433 PMC235663

[B12] Lamont R. J. Koo H. Hajishengallis G. (2018). The oral microbiota: dynamic communities and host interactions. Nat. Rev. Microbiol. 16 (12), 745–759. doi: 10.1038/s41579-018-0089-x 30301974 PMC6278837

[B13] Nicolatou-Galitis O. Sarri T. Bowen J. Di Palma M. Kouloulias V. E. Niscola P.. (2013). Systematic review of anti-inflammatory agents for the management of oral mucositis in cancer patients. Support Care Cancer 21 (11), 3179–3189. doi: 10.1007/s00520-013-1847-y 23702538

[B14] Nilsson R. H. Larsson K. H. Taylor A. F. S. Bengtsson-Palme J. Jeppesen T. S. Schigel D.. (2018). The UNITE database for molecular identification of fungi: Handling dark taxa and parallel taxonomic classifications. Nucleic Acids Res. 47 (D1), D259–D264. doi: 10.1093/nar/gky1022 PMC6324048 30371820

[B15] Parks D. H. Tyson G. W. Hugenholtz P. Beiko R. G. (2014). STAMP: statistical analysis of taxonomic and functional profiles. Bioinformatics 30 (21), 3123–3124. doi: 10.1093/bioinformatics/btu494 25061070 PMC4609014

[B16] Periasamy S. Kolenbrander P. E. (2010). Central role of the early colonizer *veillonella* sp. in establishing multispecies biofilm communities with initial, middle, and late colonizers of enamel. J. Bacteriol 192 (12), 2965–2972. doi: 10.1128/JB.01631-09 20154130 PMC2901697

[B17] Peters B. A. Wu J. Pei Z. Yang L. Purdue M. P. Freedman N. D. (2017). Oral microbiome composition reflects prospective risk for esophageal cancers. Cancer Res. 77 (23), 6777–6787. doi: 10.1158/0008-5472.CAN-17-1296 29196415 PMC5726431

[B18] Quast C. Pruesse E. Yilmaz P. Gerken J. Schweer T. Yarza P.. (2013). The SILVA ribosomal RNA gene database project: improved data processing and web-based tools. Nucleic Acids Res. 41 (Database issue), D590–D596. doi: 10.1093/nar/gks1219 23193283 PMC3531112

[B19] Rao D. Behzadi F. Le R. T. Dagan R. Fiester P. (2021). Radiation induced mucositis: What the radiologist needs to know. Curr. Probl Diagn. Radiol. 50 (6), 899–904. doi: 10.1067/j.cpradiol.2020.10.006 33279307

[B20] Rodríguez-Caballero A. Torres-Lagares D. Robles-García M. Pachón-Ibáñez J. González-Padilla D. Gutiérrez-Pérez J. L. (2012). Cancer treatment-induced oral mucositis: A critical review. Int. J. Oral. Maxillofac. Surg. 41 (2), 225–238. doi: 10.1016/j.ijom.2011.10.011 22071451

[B21] Sato T. Watanabe K. Kumada H. Toyama T. Tani-Ishii N. Hamada N. (2012). Peptidoglycan of actinomyces naeslundii induces inflammatory cytokine production and stimulates osteoclastogenesis in alveolar bone resorption. Arch. Oral. Biol. 57 (11), 1522–1528. doi: 10.1016/j.archoralbio.2012.07.012 22939375

[B22] Segata N. Izard J. Waldron L. Gevers D. Miropolsky L. Garrett W. S.. (2011). Metagenomic biomarker discovery and explanation. Genome Biol. 12 (6), R60. doi: 10.1186/gb-2011-12-6-r60 21702898 PMC3218848

[B23] Shimada E. Kataoka H. Miyazawa Y. Yamamoto M. Igarashi T. (2012). Lipoproteins of actinomyces viscosus induce inflammatory responses through TLR2 in human gingival epithelial cells and macrophages. Microbes Infect. 14 (11), 916–921. doi: 10.1016/j.micinf.2012.04.015 22561467

[B24] Siak P. Y. Khoo A. S. Leong C. O. Hoh B. P. Cheah S. C. (2021). Current status and future perspectives about molecular biomarkers of nasopharyngeal carcinoma. Cancers (Basel) 13 (14), 3490. doi: 10.3390/cancers13143490 34298701 PMC8305767

[B25] Sroussi H. Y. Epstein J. B. Bensadoun R. J. Saunders D. P. Lalla R. V. Migliorati C. A.. (2017). Common oral complications of head and neck cancer radiation therapy: mucositis, infections, saliva change, fibrosis, sensory dysfunctions, dental caries, periodontal disease, and osteoradionecrosis. Cancer Med. 6 (12), 2918–2931. doi: 10.1002/cam4.1221 29071801 PMC5727249

[B26] Stokman M. A. Spijkervet F. K. Boezen H. M. Schouten J. P. Roodenburg J. L. de Vries E. G. (2006). Preventive intervention possibilities in radiotherapy- and chemotherapy-induced oral mucositis: results of meta-analyses. J. Dent. Res. 85 (8), 690–700. doi: 10.1177/154405910608500802 16861284

[B27] Stokman M. A. Spijkervet F. K. Burlage F. R. Dijkstra P. U. Manson W. L. de Vries E. G.. (2003). Oral mucositis and selective elimination of oral flora in head and neck cancer patients receiving radiotherapy: a double-blind randomised clinical trial. Br. J. Cancer 88 (7), 1012–1016. doi: 10.1038/sj.bjc.6600824 12671696 PMC2376383

[B28] Trotti A. Bellm L. A. Epstein J. B. Frame D. Fuchs H. J. Gwede C. K.. (2003). Mucositis incidence, severity and associated outcomes in patients with head and neck cancer receiving radiotherapy with or without chemotherapy: A systematic literature review. Radiother Oncol. 66 (3), 253–262. doi: 10.1016/s0167-8140(02)00404-8 12742264

[B29] Tuominen H. Rautava J. (2021). Oral microbiota and cancer development. Pathobiology 88 (2), 116–126. doi: 10.1159/000510979 33176328

[B30] Vanhoecke B. De Ryck T. Stringer A. Van de Wiele T. Keefe D. (2015). Microbiota and their role in the pathogenesis of oral mucositis. Oral. Dis. 21 (1), 17–30. doi: 10.1111/odi.12224 24456144

[B31] Villa A. Sonis S. T. (2015). Mucositis: pathobiology and management. Curr. Opin. Oncol. 27 (3), 159–164. doi: 10.1097/CCO.0000000000000180 25774860

[B32] Washio J. Sato T. Koseki T. Takahashi N. (2005). Hydrogen sulfide-producing bacteria in tongue biofilm and their relationship with oral malodour. J. Med. Microbiol. 54 (Pt 9), 889–895. doi: 10.1099/jmm.0.46118-0 16091443

[B33] Washio J. Shimada Y. Yamada M.. (2014). Effects of pH and lactate on hydrogen sulfide production by oral veillonella spp. Appl. Environ. Microbiol. 80 (14), 4184–4188. doi: 10.1128/AEM.00606-14 24795374 PMC4068660

[B34] Wijers O. B. Levendag P. C. Harms E. R. Gan-Teng A. M. Schmitz P. I. Hendriks W. D.. (2001). Mucositis reduction by selective elimination of oral flora in irradiated cancers of the head and neck: A placebo-controlled double-blind randomized study. Int. J. Radiat. Oncol. Biol. Phys. 50 (2), 343–352. doi: 10.1016/s0360-3016(01)01444-4 11380220

[B35] Xia Y. Sun J. Chen D. G. (2018). “Power and sample size calculations for microbiome data,” in Statistical analysis of microbiome data with r. ICSA book series in statistics (Singapore: Springer), 129–166. doi: 10.1007/978-981-13-1534-3_5

[B36] Xu Y. Teng F. Huang S. Lin Z. Yuan X. Zeng X.. (2014). Changes of saliva microbiota in nasopharyngeal carcinoma patients under chemoradiation therapy. Arch. Oral. Biol. 59 (2), 176–186. doi: 10.1016/j.archoralbio.2013.10.011 24370189

[B37] Yamashita Y. Takeshita T. (2017). The oral microbiome and human health. J. Oral. Sci. 59 (2), 201–206. doi: 10.2334/josnusd.16-0856 28637979

[B38] Zhang S. Kong C. Yang Y. Cai S. Li X. Cai G.. (2020). Human oral microbiome dysbiosis as a novel non-invasive biomarker in detection of colorectal cancer. Theranostics 10 (25), 11595–11606. doi: 10.7150/thno.49515 33052235 PMC7545992

[B39] Zhu X. X. Yang X. J. Chao Y. L. Zheng H. M. Sheng H. F. Liu H. Y.. (2017). The potential effect of oral microbiota in the prediction of mucositis during radiotherapy for nasopharyngeal carcinoma. EBioMedicine 18, 23–31. doi: 10.1016/j.ebiom.2017.02.002 28216066 PMC5405060

